# ATP1A3 as a target for isolating neuron‐specific extracellular vesicles from human brain and biofluids

**DOI:** 10.1002/alz.088090

**Published:** 2025-01-09

**Authors:** Zhengrong Zhang, Yang You, Nadia Sultana, Maria Ericsson, Yuka A Martens, Min Sun, Takahisa Kanekiyo, Seiko Ikezu, Tsuneya Ikezu, Scott A Shaffer

**Affiliations:** ^1^ Mayo Clinic Florida, Jacksonville, FL USA; ^2^ Mayo Clinic, Jacksonville, FL USA; ^3^ University of Massachusetts Chan Medical School, Worcester, MA USA; ^4^ Harvard Medical School, Boston, MA USA; ^5^ Nanoview Biosciences, Boston, MA USA; ^6^ University of Massachusetts Medical School, Worcester, MA USA

## Abstract

**Background:**

Neuron‐derived extracellular vesicles (NDEVs) are a valuable resource for understanding brain conditions and discovering neurodegenerative diseases biomarkers, notably Alzheimer’s disease (AD). Recent interest focuses on capturing neuron‐specific EVs from patient‐derived samples, characterizing their contents as a pathological reflection of the central nervous system (CNS). Our recent study identified ATPase Na^+^/K^+^ Transporting Subunit Alpha 3 (ATP1A3) as a prevalent neuron‐specific EV marker specifically expressed in brains. This study systematically analyzes neuronal EVs to assess the specificity of ATP1A3 to NDEVs and its potential as a target for NDEV pulldown from accessible biofluids for disease monitoring. Additionally, ATP1A3 is compared with other proposed NDEV markers.

**Method:**

We applied immunoelectron microscopy to detect ATP1A3, L1 cell adhesion molecule (L1CAM) and neural cell adhesion molecule 1 (NCAM1) in EVs isolated from iPSC‐derived excitatory neurons, brain tissue, cerebrospinal fluid (CSF) and plasma. Neuronal EV enrichment was achieved through immunoaffinity isolation, utilizing anti‐ATP1A3, L1CAM and NCAM1 antibodies separately. Evaluation involved quantitative mass‐spectrometry, immunoblotting and ELISA. To compare the enrichment of neuronal markers, EVs isolated from CSF and plasma samples were analyzed using ExoView and Nanoimager. Additionally, we investigated the potential of ATP1A3^+^ plasma EVs as AD biomarker by comparing the value of amyloid‐beta peptide (Aβ) in ATP1A3^+^ plasma EVs measured by Nanoimager and other plasma AD biomarkers as determined by SIMOA.

**Result:**

ATP1A3, identified as a neuron‐specific protein, exhibits substantial enrichment in NDEVs isolated from induced human neurons, brain tissue, CSF, and plasma samples, surpassing NCAM1 or L1CAM. Both single‐ and bulk‐EV analysis consistently demonstrate a higher enrichment of ATP1A3 associated EVs in human samples. Immunoprecipitation of ATP1A3^+^ EVs from human brain tissues reveals superior neuronal cell‐type specificity over NCAM1^+^ and L1CAM^+^ EVs by label‐free mass spectrometry. Increased Aβ levels are observed in ATP1A3^+^ EVs derived from CSF and plasma of AD cases by SIMOA. Moreover, Aβ^+^ populations in ATP1A3^+^ EVs from plasma can distinguish AD from mild cognitive impairment and control cases via Nanoimager compared to the conventional quantification of AD markers.

**Conclusion:**

Our findings demonstrate that ATP1A3 as a promising target for isolating NDEVs from biofluids, offering potential diagnostic advancements in neurological research.

